# Effect of Low-Frequency Vibration on Muscle Response under Different Neurointact Conditions

**DOI:** 10.1155/2019/1971045

**Published:** 2019-01-03

**Authors:** Chaofei Zhang, Wenjun Wang, Dennis Anderson, Sishu Guan, Guofa Li, Hongyi Xiang, Hui Zhao, Bo Cheng

**Affiliations:** ^1^State Key Laboratory of Automotive Safety and Energy, Department of Automotive Engineering, Tsinghua University, Beijing 100084, China; ^2^Center for Advanced Orthopedic Studies, Beth Israel Deaconess Medical Center, Boston, MA 02215, USA; ^3^Chongqing Key Laboratory of Vehicle/Biological Crash Security, Department 4th, Institute of Surgery Research, Daping Hospital, Third Military Medical University, Chongqing 400042, China; ^4^Institute of Human Factors and Ergonomics, College of Mechatronics and Control Engineering, Shenzhen University, Shenzhen 518060, China

## Abstract

Stretch reflex is an important factor that influences the biomechanical response of the human body under whole-body vibration. However, there is a lack of quantitative evaluation at lower frequencies. Thus, the aim of this study was to investigate the effects of vibration on the stretch reflex and, in particular, to explore the quantitative relationship between dynamic muscle responses and low-frequency vibrations. The gastrocnemius muscle of 45 Sprague-Dawley rats was dissected. Sinusoidal vibrations of five discrete frequencies (2~16 Hz) with peak-to-peak amplitudes of 1 mm were applied to the gastrocnemius muscles with 2 mm or 3 mm prelengthening. Variables including dynamic muscle force, vibration acceleration, and displacement were recorded in two conditions, with and without the stretch reflex. Results showed that the dynamic muscle forces decreased by 20% on average for the 2 mm prelengthening group after the stretch reflex was blocked and by 24% for the 3 mm prelengthening group. Statistical analysis indicated that the amplitude of dynamic muscle force in the “with stretch reflex” condition was significantly larger than that in the “without stretch reflex” condition (*p* < 0.001). The tension-length curve was found to be a nonlinear hysteresis loop that changed with frequency. The phase difference between the dynamic muscle force and the length change was affected significantly by vibration frequency (*p* < 0.01), and the minimum frequency was 4–8 Hz. Experimental results of this study could benefit musculoskeletal model by providing a theoretical support to build a stretch reflex model for low-frequency vibration.

## 1. Introduction

Muscle fatigue caused by prolonged driving is an important factor affecting driving comfort and resulting in lower back pain [1]. Whole-body vibration exposure plays a key role in producing muscle fatigue and accelerating the fatigue process [2, 3]. Shinohara [4] synthesized several findings about the effects of prolonged vibration on muscle activity and revealed that prolonged vibration modulated muscle activity, which led to reduced peak force during maximal contractions and altered force fluctuations.

Several studies have examined the effect of muscle contractions on whole-body vibration response. Huang and Griffin [5] and Nikooyan and Zadpoor [6] reported that the activation of muscles could have a significant effect on human mechanical response under whole-body vibration. However, activation levels were set as constant values in their models, which is inconsistent with the actual situation. Kitazaki and Griffin [7] used a human finite element model with a muscle module to examine vibration comfort. Electromyography (EMG) data was used as activation inputs when predicting muscle response. However, EMG must be measured before simulation, and the quantitative relation between EMG and muscle activation is more complex in actual conditions. Brown et al. [8] and Meng et al. [9] found that the response of drivers' lumbar muscle forces reached a peak in the resonance frequency bands of the human biomechanics system (2–25 Hz). These studies suggested that human biomechanical response, especially for the muscle activity, was significantly affected by frequency of whole-body vibration.

Vibration can cause muscle lengthening and shortening, potentially resulting in increased muscle tension due to a stretch reflex [10]. Souron et al. [11] reported that a training period of local vibration was efficient in improving muscular performance. Stretch reflex was proposed as an important factor that influences the biomechanical response of the human body under whole-body vibration [12].

Human experiment had been conducted to examine the effect of vibration frequency and amplitude on the stretch reflex. Ritzmann et al. [10] reported that vibration-induced stretch reflex increased EMG activity after the elimination of motion artifacts. Miles et al. [13] reported two different stretch reflex responses, which are short-latency reflex evoked by high-frequency stretches and long-latency reflex evoked by slower stretches. Wakeling et al. [14] found increasing EMG activity and damping when the vibration frequency was close to the natural frequency of the soft tissue. Bosco et al. [15], Rittweger [16], and Cochrane [17] found increased muscle activity and power after whole-body vibration and suggested a potential benefit over traditional forms of resistive exercise. Zaidell et al. [18] also found increased muscle activation during whole-body vibration with a frequency of 25 Hz and 50 Hz and explained it with muscle modulation under tonic vibration reflex.

However, most of the above studies focused on high-frequency oscillations (over 25 Hz), while the vibration frequency in driving situations is mostly below 20 Hz. And biomechanical response such as spine loading and muscle forces which are closely related to driving comfort and fatigue had not been analyzed yet. Musculoskeletal model [19, 20] was an alternative way to calculate muscle force and joint loading which used bunches of muscle fascicles to model muscle groups. Thus, an assumption was made that the human muscle response under whole-body vibration could be modelled using the combination of many muscle fascicle responses under localized vibration.

Animal experiment is an effective method used in previous studies to analyze the mechanism of stretch reflex of a single muscle [8]. Matthews [21–23] studied the relationship between muscle tension and muscle length in different stretching velocities on decerebrate cats. Factors that could influence the stretch reflex, such as muscle length, stimulating style, and motor nerve inhibition, were analyzed. Roberts [24] studied the hysteresis loop of tension against length plots when sinusoidal fluctuating tensions were applied to the soleus muscle of decerebrate cats, suggesting that there was damping in the process of stretching. However, the period in his study is from 0.7 to 16.5 seconds; the highest frequency is 1.4 Hz that is not a common real-world driving condition. Günther et al. [25] noticed the damping in high-frequency oscillations and researched its mechanical characteristics by performing experiments on a piglet.

To date, various studies have addressed the importance of the muscle stretch reflex on human vibration responses. However, the quantitative effect of the stretch reflex induced by low-frequency (2~20 Hz) vibration on muscle response has not been sufficiently analyzed in previous studies, which is a common real-world condition and important for the human musculoskeletal model. It is also suggested that the stretch reflex responses vary with different stretch frequencies, amplitudes [26], or muscle lengths. Thus, the hypothesis of this study was that the stretch reflex plays an important role in muscle vibration response in the low-frequency range on driving condition, and the vibration frequency and initial muscle length will significantly affect the muscle response. This experimental study is aimed at (1) examining the stretch response differences between muscles with/without stretch reflex arc at different vibration frequencies and initial muscle length conditions and (2) exploring the quantitative relationship between muscle responses and low-frequency vibrations.

## 2. Methods

### 2.1. Experiment Configuration

**Figure 1:**
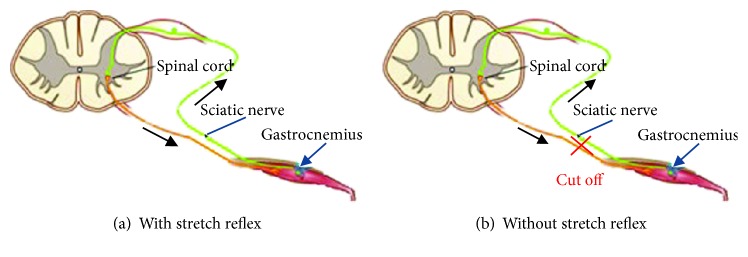
Two experimental conditions.

This study was approved by the Animal Welfare Committee, Third Military Medical University of China. Experiments were performed to examine the reflex response to sinusoidal stretching of the gastrocnemius muscles of the right hind limb of 45 male rats (225 ± 20 g, 10 ± 1 week). Decerebration was conducted by cutting the spinal cord between T9 and T12 of each of the 45 rats before experimentation. Dynamic muscle response force, muscle length, and vibration acceleration were recorded in two conditions: “with stretch reflex” (WSR) condition (Figure 1(a)), meaning the stretch reflex arc was intact for the lower-extremity muscles, and “without stretch reflex” (WOSR) condition (Figure 1(b)), meaning the stretch reflex arc was blocked by cutting off the sciatic nerve after experimentation in the WSR condition.

**Figure 2:**
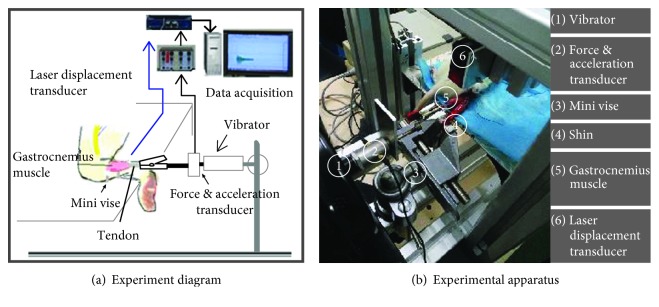
Experiment configuration.

The animal experiment diagram is presented in Figure 2(a). The gastrocnemius muscle was separated and tied to a vibrator. The lower part of the shin was fixed in a vise. Piezoelectric transducers were used to record the dynamic muscle force with the vibration stretching and vibration acceleration. A laser displacement transducer was used to measure the change in muscle length.

### 2.2. Surgical Procedure

The surgical procedures were performed under general anesthesia (ether, inhalational anesthesia). The nerves and muscles of the hind limbs were prepared, and a laminectomy was performed between T9 and T12 in preparation for later spinalization; see Figure 2(b). A few trails were conducted to explore the location of spinalization. Results indicated that the best location was between T9 and T12. If the location was too high, a serious spinal injury would occur, and if the location was too low, the stretch reflex arc would be cut off. After spinalization, the ether was removed, and the rat would regain consciousness. The effectiveness of the operation was proven by the free movement of the front legs and paralysis of the hind legs. The nerve connection between the brain and the lower extremities was cut off, and the rat was ensured to be alive.

Then the rat was anesthetized again, and its right hind limb was extensively dissected (Figure 2(b)). All the other nerves (femoral nerve, distal branches of the sciatic, obturator nerve, and hamstring nerve), except for those to the gastrocnemius muscles, were denervated. The ipsilateral gastrocnemius muscles were freed from their surrounding tissue. The end of the hind calcaneus bone was cut to keep a piece of bone to leave the tendon of the gastrocnemius muscle intact. The muscle slack length after surgery was measured.

The rat was then mounted in a stereotaxic frame (Figure 2(b)). The other three legs besides the right hind limb were fixed with adhesive tape. The shin bone of the right hind limb was held in a bench vise. The distal tendon of the gastrocnemius muscle was tied to a vibrator using an alligator clip.

### 2.3. Experimental Procedure

To investigate the effect of initial muscle length on the response, the decerebrate rats were divided into two prelengthening groups, which are 2 mm or 3 mm longer than the slack length of the muscle. Measurement results before the surgical procedure showed that the gastrocnemius lengthened 7 mm corresponding to an ankle dorsiflexion of approximately 135°. Therefore, we ensured that the length of the gastrocnemius would not exceed the physical limitation during the vibration test with a peak-to-peak amplitude of 1 mm.

Sinusoidal vibrations were applied along the longitudinal axis of the muscle. Five discrete frequencies (2, 4, 8, 12, and 16 Hz) were employed. The peak-to-peak amplitude in stretching was 1 mm for all frequencies. Each frequency was tested twice in both the WSR and WOSR conditions. In total, each rat experienced 22 tests (5 × 2 + 1 for trial 1 and 5 × 2 + 1 for trial 2). To make sure the quality of measurement, we kept recording until 10 stable periods signals have been measured and then we stopped recording the data for a test. According to our recording, the duration for the test is 35~40 s.

Because the gastrocnemius muscle was repeatedly stretched using different frequencies vibration, it may cause muscle fatigue which would affect the result of muscle response. To exclude the effect of repeated stretching, a 15 s break was added between each adjacent test for recovery. Moreover, an additional test with the same frequency as the first test in the trial was performed to examine whether the muscle was tired. No significant decline of muscle response force was observed in this additional test as compared to the first test in the same trial. This strategy ensured the exclusion of the fatigue effect on muscle response. Since we have already excluded the fatigue effect, the experiment did not use a random order.

Another important influencing factor was spinal shock in which all neurological activity was lost. Nesmeyanova [27] found a normal electrical response of the soleus muscle in spinal patients with clonus compared with absence of clonus after spinal cord injury. However, it was difficult to measure the spinal shock level quantitatively. To ensure normal response after spinal injury, we proposed the following method to avoid or mitigate the influence of spinal shock. After spinalization, the anesthesia was removed and a piece of gauze was used to stanch bleeding. After the rat regained consciousness, if the rat climbed forward or in a circle with the two front legs and the two hind legs seemed powerless because of a loss of brain control, this meant that the rat had recovered partially or totally from the spinal shock.

### 2.4. Data Progress

In total, 45 rats were involved in the experiment, 15 in the 2 mm prelengthening group and 14 in the 3 mm prelengthening group. The other 16 rats either died in surgery or failed to yield useful results, typically because of the vagaries of the decerebrate preparation rather than the occasional failure of the recording operation. A zero-phase 8th-order Butterworth low-pass filter with a cut-off frequency of 25 Hz was used for data filtering.

The system inertial mass was identified prior to the tests without muscle connection. The inertial mass included the mass of an alligator clip, a laser reflection plate for length recording, and the connection bolt to the acceleration transducer. Sinusoidal signals of 5 Hz and 10 Hz were provided by the vibrator. The force *F*_inertial_ and the acceleration *a* were recorded. The system inertial mass *m*_system_ can be computed according to

(1) $$\begin{array}{c} m_{system}=\frac{F_{inertial}}{a}. \end{array}$$

It was found that the system inertia mass was about 34.8 g. Therefore, the system inertial force can be calculated if the vibration acceleration is known. The inertial force was subtracted from the measured forces to obtain the dynamic muscle force.

**Figure 3:**
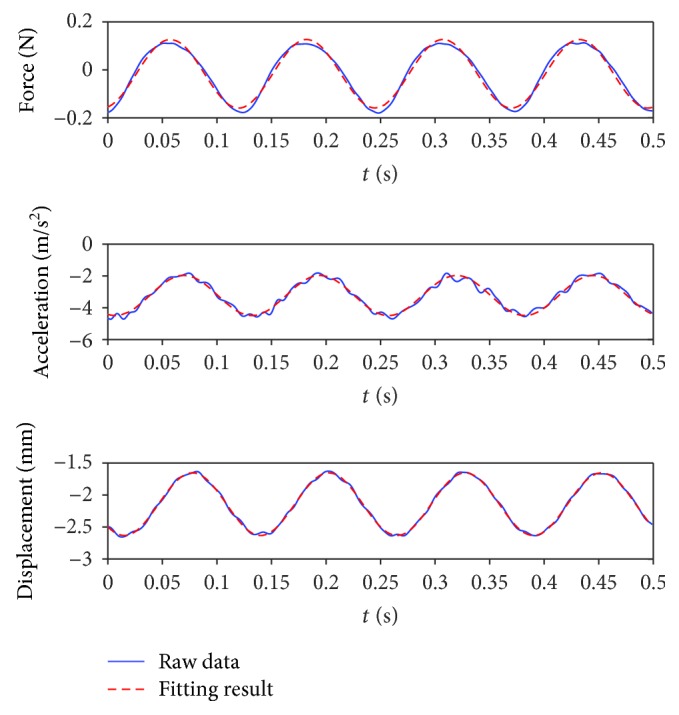
Typical example of raw data and fitting result at 8 Hz with stretch reflex under 2 mm prelengthening condition.

Ignoring the nonlinear factors, a three-parameter model was used to fit the time history curve of the experimental data with the least squares method; see equation (2).

(2) $$\begin{array}{c} g\left(t\right)=A\cdot \sin\left(2\pi ft+\varphi \right)+C, \end{array}$$

where *g*(*t*) is the time-dependent experimental data, *f* is the corresponding vibration frequency, *A* is the identification amplitude, *φ* is the identification phase, and *C* is the identification shift. An example of fitting results is shown in Figure 3. The muscle force presented here was the dynamic muscle force with the change in the muscle length. A negative value represents a decrease in the muscle force.

The phase difference between the dynamic muscle force and the muscle length change was calculated as follows:

(3) $$\begin{array}{c} ∆\varphi =\varphi_{f}-\varphi_{l}. \end{array}$$

Here, *∆φ* is the phase difference, and *φ*_*f*_ and *φ*_*l*_ are, respectively, the phase of dynamic muscle force and muscle length identified with the least squares method according to equation 2.

To exclude the effect of the individual difference, a normalization method was proposed to further analyze the force data for each rat as follows:

(4) $$\begin{array}{c} {\overset{¯}{F}}_{WOSR}=\frac{F_{WOSR}}{F_{WSR}}, \end{array}$$

where  *F*_WOSR_ and *F*_WSR_ are the identified amplitude of dynamic muscle force measured in the WOSR and WSR conditions, respectively, and ${\overset{¯}{F}}_{WOSR}$ is the normalized amplitude for the WOSR condition.

In addition, to quantify the biomechanical properties of the hysteresis loops of tension length, the stiffness and viscoelasticity were estimated as follows:

(5) $$\begin{array}{c} F=k\cdot l+c\cdot \dot{l}, \end{array}$$

where *F* is the muscle force, *k* is the stiffness, *c* is the damping coefficient, and *l* is the muscle length.

### 2.5. Statistical Analysis

Three-way analysis of variance (ANOVA, 2 × 2 × 5) was conducted to analyze the significance of stretch reflex, muscle initial length, and vibration frequency for muscle response force. Then a post hoc Tukey-Kramer test was used to compare the significance between each level. Two-way ANOVA (2 × 5) was further conducted for the normalized muscle force ${\overset{¯}{F}}_{WOSR}$. In addition, the significance of the phase difference was also explored using three-way ANOVA (2 × 2 × 5). The values *p* < 0.05 were considered statistically significant. Statistical analysis was done using the MATLAB R2017a Statistics Toolbox (The MathWorks Inc., Natick, Massachusetts, United States).

## 3. Results

### 3.1. Effect of Stretch Reflex

**Figure 4:**
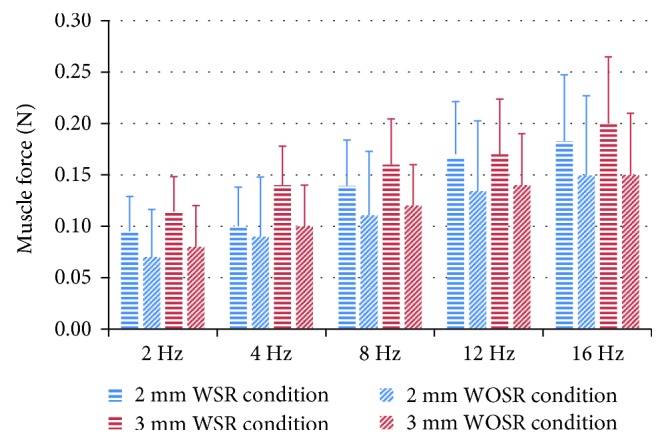
Comparison of muscle response force with/without stretch reflex for 2 mm and 3 mm prelengthening group.

The amplitudes of dynamic muscle force in the 2 mm and 3 mm prelengthening groups are summarized in Figure 4. It is clearly shown that the muscle response force with stretch reflex was larger than that without stretch reflex at every frequency for both 2 mm and 3 mm prelengthening groups.

Data analysis results (Table 1) indicated that the muscle response force decreased by 20% on average after the stretch reflex arc was blocked for the 2 mm prelengthening group and by 24% for the 3 mm prelengthening group. The statistical analysis result of three-way ANOVA for the muscle force is presented in Table 2. As we can see from Table 2, compared with WSR condition, the muscle force had a significant reduction (*F* (1,14) = 25.21, *p* = 0.000) at WOSR condition. Three-way ANOVA was also applied to phase difference, and result showed that there is no significance between WSR and WOSR conditions (*F* (1,14) = 0.86, 0 = 0.3357).

### 3.2. Effect of Initial Length

**Figure 5:**
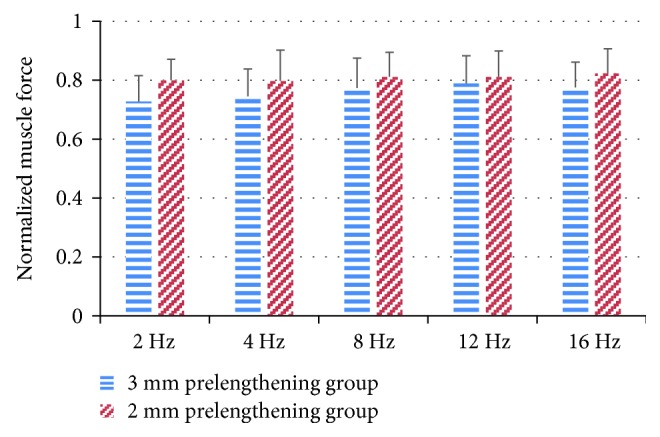
Comparison of normalized muscle response force ${\overset{¯}{F}}_{WOSR}$ between 2 and 3 mm prelengthening group.

The average dynamic muscle force in the 3 mm prelengthening group was larger than that of the 2 mm prelengthening group for all frequencies (Figure 4). However, there was no significant difference between 2 mm and 3 mm prelengthening group (Table 2, *F* (1,14) = 2.06, *p* = 0.1522). Normalized muscle force ${\overset{¯}{F}}_{WOSR}$ was also analyzed (Figure 5), and two-way ANOVA showed a significant difference between 2 mm and 3 mm prelengthening group (*F* (1,13) = 9.1, *p* = 0.003). In addition, the effect of the initial length on the phase difference was investigated, and no significant difference was found for both the WSR and the WOSR conditions (*F* (1,14) = 2.02, *p* = 0.1561).

### 3.3. Effect of Vibration Frequency

As can be seen from Figure 4, the dynamic muscle force increased with increasing vibration frequency in both prelengthening groups. Note that the muscle force of the WOSR condition also had an increasing trend with increasing frequency. Three-way ANOVA analysis showed that the vibration frequency had a significant effect on the amplitude of dynamic muscle force (Table 2, *F* (1,14) = 25.21, *p* = 0.000). The post hoc Tukey-Kramer test showed that the lower frequency (2 Hz) and higher frequency (16 Hz) had a significant effect on the dynamic muscle force, while the middle frequency (8 Hz) did not show any significance. However, the frequency had no significant effect on the normalized muscle force ${\overset{¯}{F}}_{WOSR}$ (*F* (4,52) = 0.95, *p* = 0.4377).

**Figure 6:**
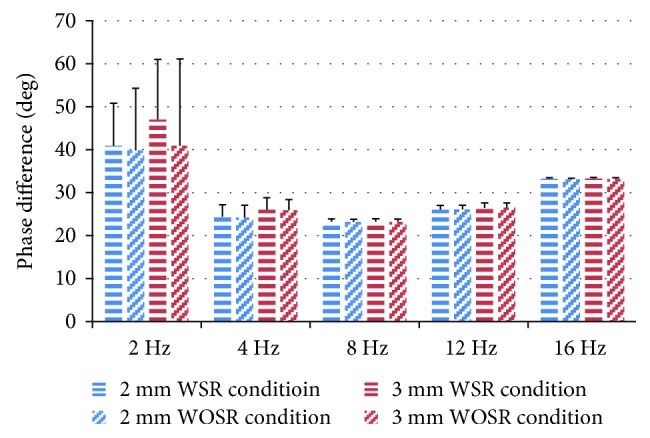
Phase difference between dynamic muscle force and length change for 2 mm and 3 mm prelengthening group.

Phase differences between the dynamic muscle force and the length change were also analyzed (see Figure 6). The force had a phase lead compared with the length change. The phase difference decreased sharply at first and then increased slowly with increasing frequency for the 2 mm and 3 mm prelengthening groups. It reached the lowest value at approximately 8 Hz at approximately 20°. Surprisingly, the variance of the phase at 2 Hz was extremely large among the tested rats. As the frequency increased, the variance reduced rapidly and almost disappeared at 8 Hz. Significance was observed from three-way ANOVA analysis (*F* (4,56) = 73.73, *p* = 0.000). The post hoc Tukey-Kramer test showed that 2 Hz and 16 Hz significantly differed with the other three groups at *p* < 0.05; the middle frequencies (4~12 Hz) were not significantly different with each other.

### 3.4. Tension-Length Curves

**Figure 7:**
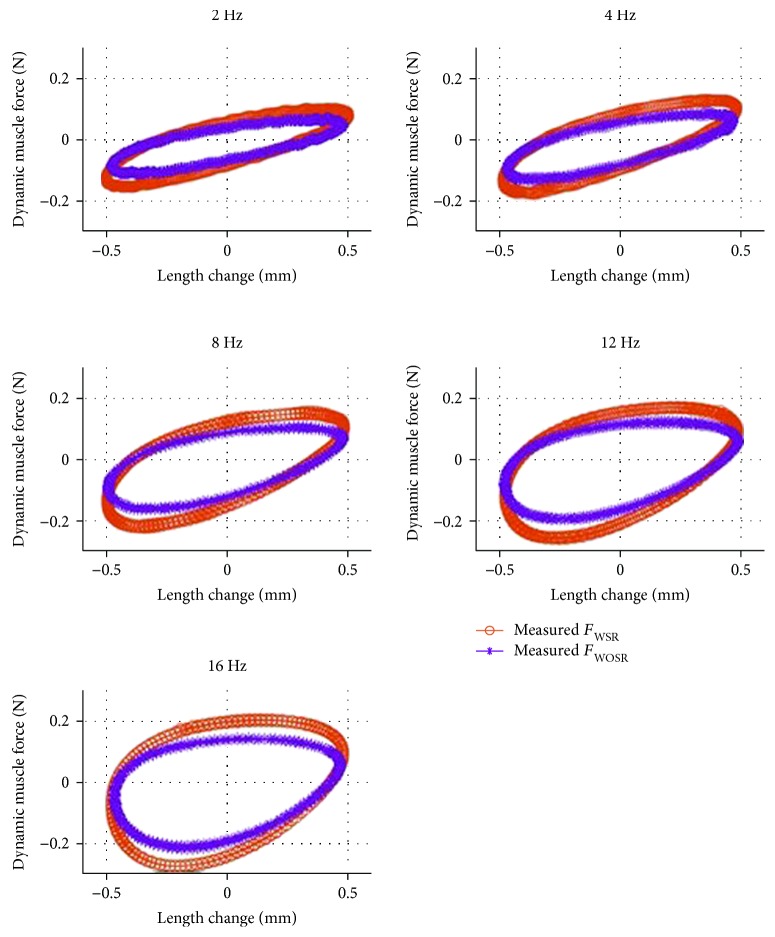
Representative muscle force vs. length curve (2 mm prelengthening group).

**Figure 8:**
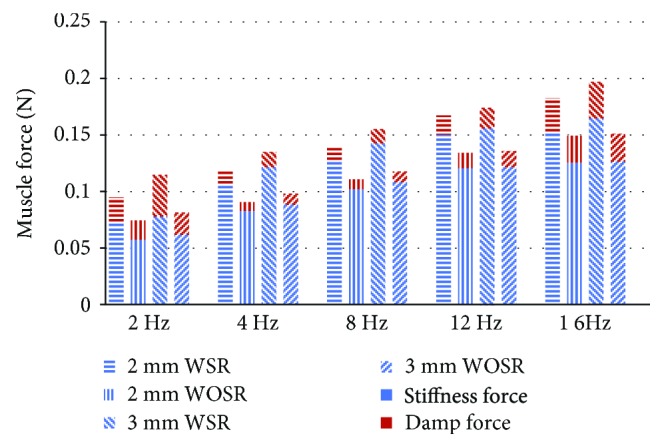
Identification of average stiffness force and damp force of the hysteresis loops.

The relationship between muscle force and muscle length for the 2 mm prelengthening group is shown in Figure 7. The blue and red lines represent the dynamic muscle force of the WSR and WOSR conditions, respectively. The force-length curve shows a clockwise loop, and its shape is substantially an ellipse. It indicates that both damping and stiffness factors were included in the muscle biomechanical system. The distribution of the stiffness and damp forces calculated according to equation 5 is presented in Figure 8. It was shown that the damp force was highest at 2 Hz and then had a linear increase from 4 to 16 Hz. Nonlinear increase of the stiffness was observed with frequency. Consistent with the dynamic muscle force, the stiffness was also decreased after the stretch reflex arc was blocked.

## 4. Discussion

This study investigated the gastrocnemius muscle force response to vibration stretching of 2–16 Hz using decerebrate rats. Significant reductions in dynamic muscle force response were observed with the elimination of stretch reflex (*p* < 0.001). This is consistent with the findings of previous studies using nerve inhibition methods, such as sectioning of the reflex arc nerve and nerve anesthesia. Roberts [24] observed an increase of tension with the stretch reflex and redefined stretch reflex as an increase of muscle stiffness. Serres et al. [28] published similar results using the triceps surae muscles of decerebrate cats. After sectioning the L5 to S2 dorsal roots, muscle response force decreased significantly for low and high levels of background force.

Although these studies reached a similar conclusion that muscle response to vibration is significantly reduced after the stretch reflex arc is disrupted, few studies have quantified the difference between the WSR and WOSR conditions. This study found an over 20% reduction after the stretch reflex arc was blocked, which mainly depended on the stretching magnitude and frequency. It also showed that a greater prelengthening might lead to a higher muscle force at lower frequencies such as at 2–8 Hz, but not at 12–16 Hz. Though both higher frequency and greater prelengthening could lead to a higher muscle force, prelengthening was the dominant factor at low frequency, so the muscle force was greater at higher prelengthening condition. While frequency was the dominant factor at high frequency, the muscle force seemed the same for the two prelengthening condition tested in our study. However, there is no significance between the two prelengthening conditions for muscle force (*F* (1,14) = 2.06, *p* = 0.1522), while a significant difference was observed for normalized muscle force ${\overset{¯}{F}}_{WOSR}$ (*F* (1,13) = 9.1, *p* = 0.003).

The obtained results showed that the tension-length curve was a clockwise loop and its shape was substantially an ellipse. Roberts [24] researched rhythmic excitation of the stretch reflex using the soleus muscle of decerebrate cats. Rhythmic excitation of several frequencies and amplitudes was used to stretch the muscle. It was found that plots of tension against length showed a clockwise hysteresis loop consistent with the results found in this study. Jansen and Rack [29] also studied the stretch reflex with the soleus muscle of cerebrate cats by sinusoidal stretching of the soleus muscle at various frequencies and amplitudes. Similarly, clockwise elliptical hysteresis loops were observed at some frequencies with a 1 mm peak-to-peak amplitude. It was also reported that when the stretch amplitude was increased to 3.8 mm (peak-to-peak), the tension-length ran clockwise. The effect of stretch amplitude on the tension-length hysteresis loop needs to be studied in future work.

Further analysis for hysteresis loop indicated that the stiffness had a nearly linear increase from 2 Hz to 12 Hz and then stayed stable from 12 Hz to 16 Hz (Figure 8). The damp force was highest at 2 Hz and then had a linear increase from 4 to 16 Hz. But when considering damp coefficient, that is the division of damp force and stretch velocity, a downtrend was observed from low to high frequencies. The result of the hysteresis loop indicated a nonlinear relation of dynamic muscle force and frequency, while Hasan [30] built a spindle afferent model to research response force under muscle stretch, whose parameters were the same for different stretch velocities.

In this study, the phase difference between the dynamic muscle force and the length change was calculated (see Figure 6). The large variance of phase difference at low frequencies may be related to the fact that the phase was mainly influenced by the damping force and was relatively small at low frequencies. Therefore, the phase of the muscle force would be heavily influenced by random error. The overall phase difference trend showed that the minimum value was 4~8 Hz. Furthermore, the phase difference between the WSR and WOSR conditions was negligible (*p* > 0.5), which suggested the stretch reflex had little influence on the damping factor of the muscle spindle.

The phase difference between the muscle force and length was compared with that of previous studies. Roberts [24] reported that length change lagged behind tension change by 15–20°, which is consistent with our findings. However, the phase difference was independent of the imposed frequency reported by Roberts (0.6–1.4 Hz), while the phase advance of muscle force varied with frequency (2–16 Hz) in our result. Lippold et al. [31] found an approximate 90° phase difference between the sensory discharge and the displacement record at frequencies between 4 and 15 Hz. He suggested that the muscle spindle response was greatest when velocity, rather than displacement, was maximal. Another result worth noting was the nonlinearity of the phase difference of the WOSR and WSR conditions. This phenomenon indicated that the tension-length diagram could not be attributed to frequency-dependent damping. A mathematical model based on identification method should be proposed in further study.

**Figure 9:**
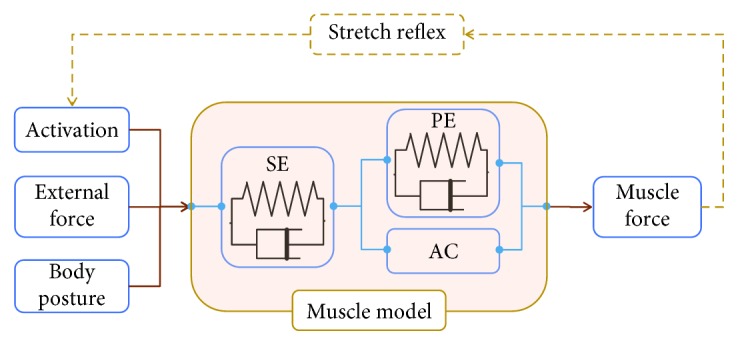
Diagram of current muscle model in solid line and potential stretch reflex module in dash line.

The results of this study could benefit musculoskeletal modeling by providing a theoretical support to build a stretch reflex model for low-frequency vibration. Frequently used muscle models include Hill muscle model [32–35], Thelen muscle model [36], and Millard muscle model [37]. The structures of the above models are similar as shown in Figure 9. External force (caused by vibration), activation (measured via EMG), and body posture or motion were model inputs, and muscle force was the model output (marked as solid lines in Figure 9). However, these muscle models could not conduct simulations when the activation was missing, because the amount of activation was difficult to measure.

There are several studies that had built linearly stretch reflex models to research the neural control and human locomotion. Geyer and Herr [38] built a reflex model to research walking using a proportional coefficient for muscle length combined with upper and lower constrains. Similarly, two proportional coefficients for angle and angle velocity were used in Eilenberg's stretch reflex model [39]. However, nonlinearity relation of phase difference is observed in this study which is consistent with Miles et al.'s [13] findings. Zhang et al. [40] and Mirbagheri et al. [41] used system identification methods to model the intrinsic properties of human arm and ankle system separately. The nonlinearity suggests that simple frequency-dependent damping (proportional method) does not really match the reality. Therefore, a more realistic muscle model incorporating stretch reflex module is needed to perform human vibration simulation based on our current work.

However, even though we have researched the effect of different low-frequency vibrations on the muscle response at different neurointact conditions and provided theoretical support for muscle model, there are still some limitations of this study: (1) although the effects of vibrational frequency and muscle length were examined in detail, the amplitude of the stretching was not considered; (2) as fatigue is a common but challenging problem in driving, the effect of different fatigue levels on muscle response needs to be considered in future studies; (3) the result of this study is based on the rat, even many studies suggest it is similar for human muscle, more validations should be done for the muscle model based on this result; (4) the relation between response under the whole-body vibration and localized vibration is very complex, further research should be conducted. We will build a new muscle model incorporating the stretch reflex module (Figure 9) based on this experimental result. More experiments considering the effect of fatigue will be conducted in the future.

## 5. Conclusion

This study explored the biomechanical response of decerebrate rats with/without stretch reflex to low-frequency vibration and described the quantitative relationship between muscle force and muscle length. Results indicated that the amplitude of muscle response force decreased by over 20% when the stretch reflex arc was blocked (*p* < 0.001). The relationship between muscle response force and muscle length was found to be a nonlinear hysteresis loop that changed with frequency (Figure 7). The phase difference between the dynamic muscle force and the change in muscle length was affected significantly by the vibration frequency (*p* < 0.05), and the minimum frequency was 4–8 Hz. Experimental results of this study demonstrated that the stretch reflex had a tremendous effect on muscle vibration response (over 20%) and could benefit musculoskeletal modeling by providing a theoretical support to build a stretch reflex model for low-frequency vibration.

## Figures and Tables

**Table 1:** Percent decrease of dynamic muscle force from WSR to WOSR condition.

(1 − (*F*_WOSR_/*F*_WSR_)) × 100%	2 Hz	4 Hz	8 Hz	12 Hz	16 Hz
2 mm prelengthening condition	20.1%	20.3%	18.9%	18.9%	17.7%
3 mm prelengthening condition	27.2%	25.5%	22.7%	21.1%	22.6%

**Table 2:** Three-way ANOVA analysis result of dynamic muscle force.

Source	df	*F*	*p*
*L* (muscle length, 2 levels)	1	2.06	0.1522
*F* (frequency, 5 levels)	4	17.74	0.000
*S* (stretch reflex, 2 levels)	1	25.21	0.000
*L* × *F*	4	0.06	0.9931
*L* × *S*	1	0.61	0.4358
*F* × *S*	4	0.11	0.9782

## Data Availability

The data used to support the findings of this study are available from the corresponding author upon request.

## Additional Points

*New and Noteworthy*. Stretch reflex is an important factor that influences the biomechanical response of the human whole-body vibration, which has not been sufficiently analyzed for low-frequency condition in previous studies. Experiment has been conducted to explore the quantitative effect of the stretch reflex induced by low-frequency vibration on muscle response, which is common in real-world condition and important for the human musculoskeletal model. Results of this study demonstrated that the stretch reflex had a tremendous effect on muscle vibration response (over 20%) at low frequencies and could benefit musculoskeletal modeling by providing a theoretical support to build a stretch reflex model for low-frequency vibration.
